# In vivo experimental study comparing alveolar ridge preservation versus guided bone regeneration after unassisted socket healing at intact and damaged sites in narrow alveolar ridges

**DOI:** 10.1002/JPER.24-0125

**Published:** 2024-07-15

**Authors:** Hae Jee Shin, Jin‐Young Park, Hsu Kuo Tien, Franz‐Josef Strauss, Jae‐Kook Cha, Jung‐Seok Lee

**Affiliations:** ^1^ Department of Periodontology Research Institute of Periodontal Regeneration, Yonsei University College of Dentistry Seoul South Korea; ^2^ Clinic of Reconstructive Dentistry University of Zurich Zurich Switzerland; ^3^ Faculty of Dentistry Universidad Finis Terrae Santiago Chile

**Keywords:** alveolar ridge augmentation, animal model, bone regeneration, bone substitutes, tooth extraction

## Abstract

**Background:**

To compare bone regeneration and dimensional alteration of alveolar ridge at intact and damaged extraction sockets after alveolar ridge preservation (ARP) and implant placement versus unassisted socket healing followed by guided bone regeneration (GBR) with simultaneous implant placement.

**Methods:**

In 6 beagle dogs, 3 types of extraction sockets in the mandible were created: (1) intact sockets, (2) 1‐wall defect sockets and (3) 2‐wall defect sockets. The sockets were allocated to undergo either (1) ARP and implant placement 8 weeks later (ARP group) or (2) GBR with simultaneous implant placement after 8 weeks of unassisted socket healing (GBR group). After an additional healing period of 8 weeks, bone regeneration and dimensional changes were evaluated radiographically and histologically.

**Results:**

GBR showed superior bone formation and greater bone gains compared to ARP, regardless of the initial extraction‐socket configuration. Although ARP maintained the preexisting alveolar ridge dimensions, peri‐implant bone defects were still detected at 8 weeks of follow‐up. Histomorphometric analyses confirmed that GBR increased dimensions of the alveolar ridge compared to baseline, and the augmentation and bone regeneration were greater with GBR than with ARP.

**Conclusion:**

Early implant placement with ARP can mitigate alveolar ridge changes in the narrow alveolar ridge. However, early implant placement with simultaneous GBR creates the conditions for enhanced bone regeneration around the implant and greater ridge augmentation compared to ARP, irrespective of the extraction‐socket configuration.

## 1 INTRODUCTION

The presence of alveolar bone of sufficient width surrounding the dental implant has traditionally been considered a prerequisite for clinical success, but recent clinical data indicate that the implant with minimal peri‐implant bone thickness can also be successful.^1^, ^2^ Alveolar ridge preservation (ARP) and guided bone regeneration (GBR) have emerged as prominent approaches for preserving and augmenting the alveolar ridge dimensions.^3^, ^4^ Dimensional collapse always occurs after tooth extraction, particularly in the buccal region, regardless of the presence of socket wall damage.^5^, ^6^ While ARP focuses on preserving the original alveolar ridge before dimensional collapse occurs, GBR aims to reconstruct such collapse from the time of initial tooth extraction and thereafter. GBR is a well‐established and documented procedure,^7^ while ARP was introduced more recently in dental practice but is supported by a growing body of preclinical and clinical data. Moreover, due to its technical simplicity and minimal invasiveness, ARP has been increasingly utilized over the past 2 decades.

ARP is a clinical procedure initially pioneered through landmark preclinical studies in dogs.^8^, ^9^, ^10^, ^11^ These studies primarily employed a single intact extraction‐socket model with a thin buccal bone plate, leading to the initial recommendation to use ARP in single‐site applications in the anterior region. However, the indications for ARP have since been expanded to encompass posterior regions ^12^, ^13^ as well as damaged sockets.^14^, ^15^, ^16^ The rationale for this broader application lies in the capacity of ARP to mitigate dimensional shrinkage both horizontally and vertically, even for sockets with wall defects.^16^ The technique provides a simplified grafting procedure as it is performed before dimensional collapse occurs, and offers clinical advantages inherent to a staged approach, including a reduction in risks associated with implant or grafting failure. Nevertheless, like any clinical procedure, ARP presents limitations alongside its advantages. In a recent clinical trial, we observed a rather widely varying amount of histologic bone regeneration, ranging from 0% to 40%, at implant sites within the grafted region.^17^ Additionally, ARP does not guarantee to reduce additional grafting procedures at the time of implant placement;^18^, ^19^ 60% of ARP sites necessitated additional augmentation through the use of supplementary GBR procedures.^20^

The clinical decision between ARP and GBR following unassisted socket healing requires thorough consideration, particularly for alveolar ridges with pathologically or dimensionally insufficient conditions. First, when suppuration limits debridement, significant baseline recession, and no bleeding is achieved for the bone,^21^ staged treatments are necessary. Second, in cases of implantation in a narrow alveolar ridge, such as in the lower anterior region, unnecessary repetition of 2 grafting procedures may occur, similar to the aforementioned 60% of ARP sites.^22^ One preclinical study comparing ARP versus implantation with GBR after spontaneous healing, demonstrated comparable volumetric changes through radiographic and digitally scanned clinical images.^23^ Nonetheless, a comprehensive and well‐informed clinical decision‐making process requires consideration of not only the external volume data but also histologic new bone formation and the implant surface area supported by osseointegration.

Therefore, the aim of the study was to compare bone regeneration and alveolar ridge changes at intact and damaged implant sites after ARP versus unassisted socket healing followed by GBR utilizing an in vivo model. This investigation specifically examined within the context of narrow alveolar ridges^24^, ^25^ with implant placement at a position of the preexisting dental root using a surgical guide template.

## 2 MATERIALS AND METHODS

### 2.1 Animals and materials

Six male beagle dogs weighing 25‐30 kg and aged 18‐24 weeks were used in this study. The animals were individually housed at a normal temperature and humidity, and provided a standardized diet. The sample size was determined based on the previous studies.^6^, ^26^ Three Rs principle (replacement, reduction, and refinement) in animal study was followed. The study design was based on the ARRIVE guideline,^27^ and its protocol including experimental units and humane endpoints, was approved by the Institutional Animal Care and Use Committee, Yonsei Medical Center, Seoul, Korea (IRB No. 2020‐0250).

Particulate type of deproteinized porcine bone minerals (DPBM; THE Graft, Purgo Biologics, Seongnam, Korea) and non‐crosslinked collagen membranes (TheCover, Purgo Biologics) were used for both ARP and GBR.^26^ To reproduce the case of implantation on a narrow alveolar ridge in a beagle model, the implants used had a diameter of 3.5 mm and a height of 8.5 mm (Anyridge, Megagen Implant, Daegu, South Korea).

### 2.2 Study design and group allocation

The study comprised 2 primary groups: (1) ARP group (*n *= 6), in which ARP was performed after tooth extraction, followed by implant placement at 8 weeks; (2) GBR group (*n *= 6), in which unassisted socket healing was allowed after tooth extraction, followed by GBR and simultaneous implant placement at 8 weeks.

A split‐mouth design was adopted, with each group being assigned to 1 unilateral alveolar ridge, while the other group was applied to the contralateral alveolar ridge.

Three distinct extraction‐socket models were involved, using protocols established in previous studies:^6^, ^26^, ^28^ (a) intact, (b) 1‐wall‐damaged (buccal wall defect) and (c) 2‐walls‐damaged (defects in both the buccal and lingual walls). Each type of extraction‐socket model was methodically allocated to the 2nd (P2), 3rd (P3), and 4th (P4) premolars to ensure even distribution.

### 2.3 Surgical protocol

All surgical procedures were performed under general anesthesia with alfaxalone (2‐3 mg/kg, IV; Jurox, Rutherford, NSW, Australia) and with isoflurane inhalation (2‐3%; Forane, Choongwae Pharmaceutical, Seoul, South Korea).

A. Tooth extraction and ARP procedures (first surgery)

**FIGURE 1:**
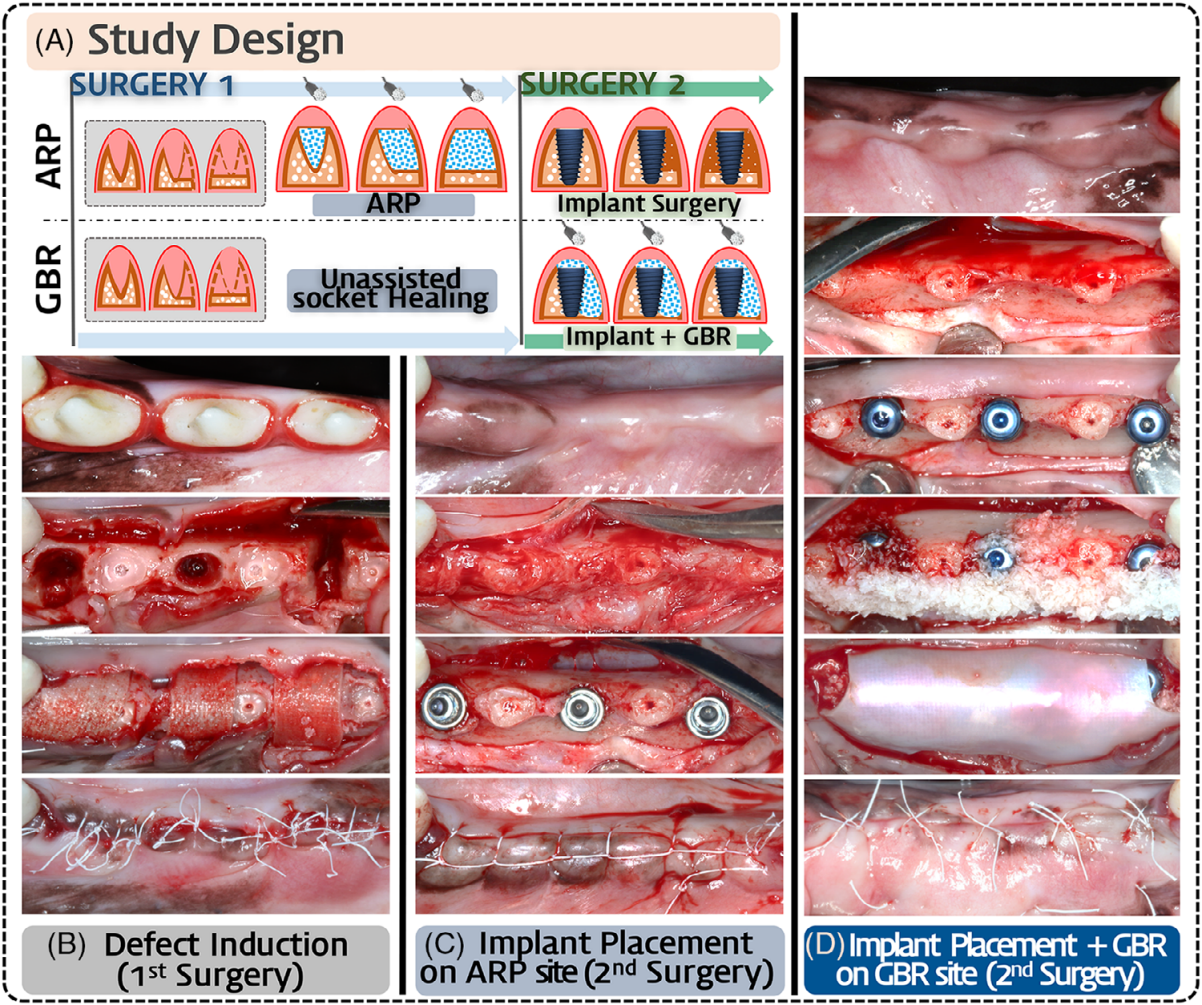
Experimental study design and clinical photographs of the surgical procedures. Timeline showing the study protocols including the respective treatment procedures and healing phases in the ARP and GBR groups (A). Mesial roots were kept as references and distal roots were extracted for experimental purposes. The 3 types of extraction‐socket models (intact, 1‐wall‐damaged and 2‐walls‐damaged) produced were treated with ARP followed by modified horizontal mattress suturing (B). After 8 weeks of healing period, the ARP group underwent implant placement followed by single interrupted suturing (C). The GBR group underwent 8 weeks of spontaneous healing after the tooth extraction, the implant was placed and simultaneous GBR was performed followed by single interrupted suturing (D).

Three premolars (P2, P3, and P4) of each unilateral mandible were hemisectioned, and the distal roots were removed using root forceps. At these sites, 3 types of standardized intact and damaged extraction sockets were produced; a whole length of the buccal bone wall or buccal/lingual walls in the sockets were surgically removed using a high‐speed rotary device with a carbide fissure bur for the 1‐wall‐damaged and 2‐walls‐damaged models. The mesial roots were decoronated at the level of bone crest and were maintained for use as the pristine control site. In ARP sites, all 3 types of extraction sockets were filled in the dimensions of pristine alveolar ridge with DPBM, and covered with the collagen membrane. The periosteal flaps were repositioned and sutured to obtain primary closure (4/0 Coated Vicryl, Ethicon, U.S). In GBR sites, the periosteal flaps were sutured right after the defect induction. Antibiotic medication and wound dressing with saline irrigation were applied for 1 week, and then, the sutures were removed (Figure 1B).

B. Fabrication of surgical guide templates

Before tooth extraction, digital scans of premolars (P2, P3, and P4) and the intact alveolar ridge were performed using an oral scanner (i700, Medit, Seoul, Korea). Subsequently, a resin jig was created based on the 3‐dimensionally printed cast. Following tooth extraction, the trajectory of the pristine dental root was manually established along the center of the extraction socket, aligning with the socket axis. This trajectory was then superimposed onto the pre‐captured scan data. The fabrication of guide templates was carried out using computer software (Exoplan, exocad GmbH, Hessen, Germany), with the position and axis of the implants planned based on information derived from the center of the preexisting extraction‐socket.

C. Implant placement and GBR procedures (second surgery)

Both ARP and GBR groups received implants after 8 weeks of healing. Full‐thickness mucoperiosteal flaps were elevated and the implants were placed in accordance with the prefabricated surgical guide templates. No cortical perforations were performed. At ARP sites, no additional bone augmentation was performed even in cases of implant dehiscence. At GBR sites, DPBM was augmented on the exposed implant surface and then covered with a collagen membrane. The periosteal flaps were repositioned and sutured, and oral antibiotics were administered. Primary closure was performed on both groups. A wound dressing was applied with saline irrigation for 1 week, and then the sutures were removed (Figure 1C,D).

D. Sample collection

All dogs were sacrificed at 8 weeks after implant placement. Intraoral scanning was performed and both alveolar ridges were retrieved for use in radiographic and histologic analyses. The obtained specimens were fixed in 10% neutral buffered formalin.

### 2.4 Micro‐computed tomography radiographic analysis

**FIGURE 2:**
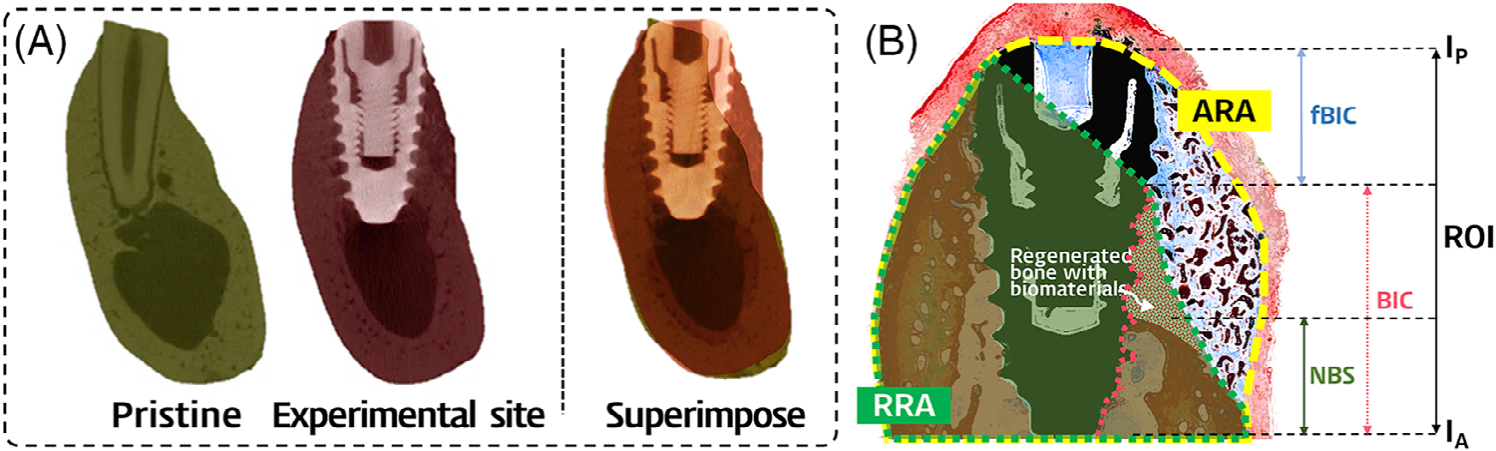
Description of radiographic and histomorphometric measurements. The proportion of alveolar preservation was calculated from the pristine mesial root (baseline) to the experimental site (post‐surgery) through the superimposition of micro‐CT images (A). Schematic diagram of the evaluation method for the histologic analyses (B). ARA, RRA, and linear measurements related to osseointegration were made on histologic slides within ROI defined from (1) the outermost margin of total tissue on both buccal and lingual aspects, and (2) perpendicular line drawn from the implant apex (IA) and implant platform (IP).

Micro‐computed tomography (micro‐CT) scanning (Skyscan 1173, Bruker) was performed (800 projections and 4 frame averages) at a resolution of 32 µm (achieved using 100 kV and 60 µA). The micro‐CT images were converted into DICOM format and transferred with the cross‐sectional slide images for morphometric evaluations using 3‐dimensional analysis software (On‐Demand3D, Cybermed). Coronal sections of the mandible were captured for the superimposition process. The most central region of both the distal experiment site (post‐surgery) and the mesial dental root (baseline) were superimposed with the aid of reference anatomical structures such as the mandibular canal and the lower border of the mandible (Figure 2A). Radiographic analysis was conducted using standard image processing software (Adobe Photoshop CS5, Adobe Systems). The region of interest (ROI) for the micro‐CT measurements was defined as the area of entire alveolar bone from the apex to the most‐crestal region. The proportion of alveolar preservation was calculated as a percentage of the ratio of the total alveolar ridge area at an experimental site to that at the corresponding pristine site.

Additionally, the ridge width of the experimental animals was measured at the mesial dental root (baseline) to delineate the extent of the narrow alveolar ridge. The ridge width was assessed at the ridge crest at pristine control sites of hemisected premolars.

### 2.5 Histological preparation and analysis

The specimen from each unilateral mandible was sectioned into 3 blocks containing 3 experimental sites that received dental implants. Bone blocks containing each site were fixed in 10% neutral buffered formalin, and then dehydrated with ethanol solutions and embedded in methyl methacrylate (Technovit 7200, Kulzer, Germany) for the production of ground sections. The most central buccolingual sections were obtained from the sites with dental implants. The ground sections were produced with a final thickness of 50 µm and then stained with Goldner's trichrome stain. Histologic slides were digitally scanned at a magnification of ×200 (Panoramic 250 Flash III), and histomorphometric analysis was performed using standard software (Adobe Photoshop CS5, Adobe Systems) by 1 experienced examiner (H.J.S.). An ROI for the histomorphometric analysis was defined using (1) the outermost margin of total tissue on both the buccal and lingual aspects, and (2) a perpendicular line drawn from the implant apex and implant platform (Figure 2B).

The following parameters were measured in the ROI:

Bone‐to‐implant contact (BIC), proportion of bone‐contacted implant surface relative to the entire implant surface at the buccal aspect.

First bone‐to‐implant contact (fBIC), the distance from the implant platform to the first bone‐to‐implant contact at the buccal aspect.

Natural bone support (NBS), the height of native bone tissue measured form the implant apex (I_A_) at the buccal aspect. (regenerated bone occupying the interspace of residual biomaterials was excluded)

Augmented ridge area (ARA), the area demarcated by the outermost line of grafted biomaterials and alveolar bone from the level of I_A._

Regenerated ridge area (RRA), the area demarcated by the outermost margin of newly formed bone and alveolar bone from the level of I_A._ (bone isolated from the defect margin was excluded)

### 2.6 Statistical analysis

All statistical analyses were performed using computer software (version 20.0, SPSS), and all parameters are presented as mean and standard deviation. The Kolmogorov‐Smirnov and Mauchly's sphericity tests were applied to evaluate the normality of the data and the sphericity assumption, respectively. Repeated‐measures analysis of variance (ANOVA) was used for intergroup (ARP and GBR groups) and intragroup (3 types of socket model) analyses. Bonferroni *p‐*value correction was applied to detect significant differences. The cutoff for significance was set as a *p‐*value of 0.05, with a modified *p‐*value of 0.016 applied in the intragroup analyses.

## 3 RESULTS

### 3.1 Clinical findings

Horizontal shrinkage could be seen at the experimental sites after unassisted socket healing (GBR group) from tooth extraction and defect creation. The damaged (defect‐induced) sites exhibited more pronounced buccolingual collapse compared to intact socket sites. However, sites in the ARP group showed reduced dimensional collapse in comparison to the above‐mentioned sites in the GBR group, irrespective of the socket configuration. After flap elevation, ARP sites showed some residual biomaterials and newly formed bone tissue were observed on the internal surface of the flap. Horizontal dehiscence defects were present in both groups, but at ARP sites, the exposed surfaces of the implant were reduced (Figure 1).

### 3.2 Micro‐CT observations

**FIGURE 3:**
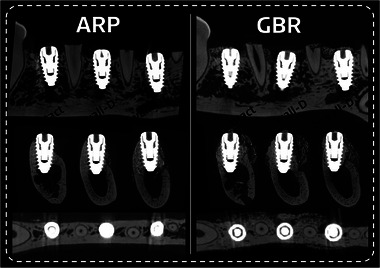
Radiographic results with micro‐CT images of the ARP and GBR groups for the 3 types of extraction‐socket configuration. Panoramic and axial views showing the 3 types of extraction‐socket configuration allocated at each distal site. Cross‐sectional micro‐CT images with 2 experimental groups were aligned according to the extent of extraction‐socket damage. The augmented dimensions were larger and the radiopacity next to the implant fixture was higher in the GBR group than in the ARP group.

Radiographs in the ARP group revealed some exposure of implant surfaces with buccal dehiscence defects. The dehiscence was largest in intact sockets, yet radiopaque granules partially covered the implant surface in the dehiscence defect area in both types of damaged sockets. Conversely, all sites in the GBR group displayed a significantly increased ARA with scattered radiopaque granules in the outermost region (Figure 3). In addition, there was no clear demarcation between the outer line of the preexisting alveolar bone and the augmented region.

### 3.3 Histologic observations

Newly formed bone contacting the most coronal area of the implant was situated higher at GBR sites than at ARP sites. The graft particles fully covered the entire implant surfaces at GBR sites, whereas ARP sites exhibited dehiscence defects that exposed the implant surfaces directly to the surrounding connective tissue. In addition, RRA was larger at the GBR sites than at the ARP sites.

At the buccal aspect, scattered unintegrated graft particles were observed over the regenerated alveolar ridge in both the ARP and GBR groups. In contrast to the buccal region, the lingual bone walls of 3 extraction‐socket models seemed less compromised than the buccal bone walls, regardless of the treatment type (ARP or GBR), even at the 2‐walls‐damaged sites.

**FIGURE 4:**
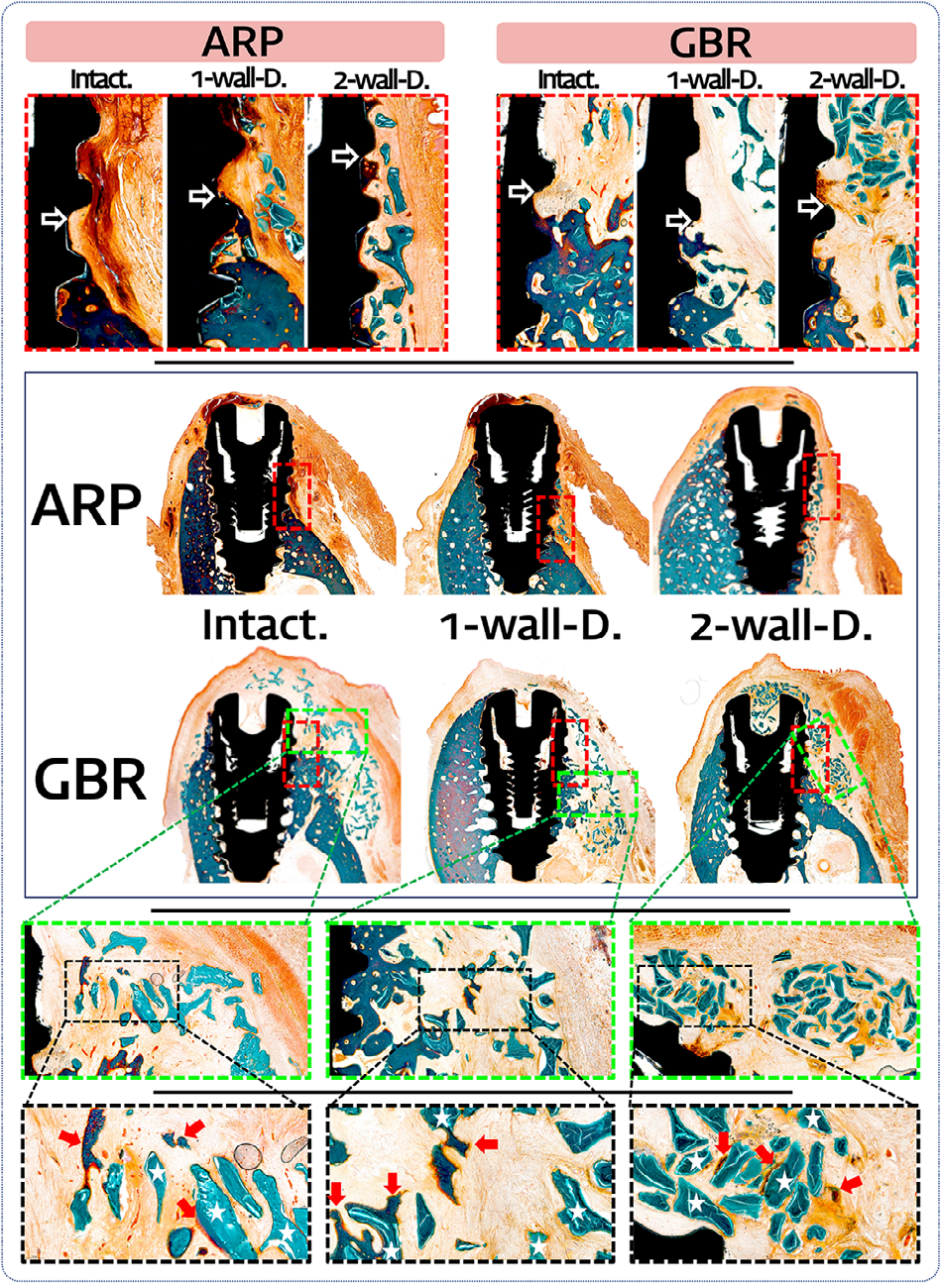
Histologic results in the ARP vs GBR groups for the 3 types of extraction‐socket configuration. Magnified views obtained from histologic sections in order of Intact, 1‐wall‐damaged and 2‐walls‐damaged socket models shown from left to right. The GBR group showed less dehiscence around implant fixture than ARP group in terms of fBIC (black arrow) results. Moreover, The GBR group showed evidence of osteogenesis in space between the biomaterial granules (asterisk*) and manifested multinucleated cells (red arrow) onto their surfaces, indicating bone‐regenerative potential within the ARA was present on the buccal aspects of each type of damaged extraction‐socket model.

Highly magnified views revealed that the outermost margin of newly formed bone at the ARP sites appeared mature, characterized by lamellar bone formation and fibrous‐encapsulated biomaterials beyond the regenerated alveolar ridge. In contrast, GBR groups exhibited an unclear margin of newly formed bone, with little evidence of osteogenesis around the residual particles of biomaterials. This suggests an ongoing phase of bone formation and remodeling processes (Figure 4).

### 3.4 Quantitative measurements of superimposed micro‐CT images and histologic slides

The mean alveolar ridge widths of the pristine sites were 4.59 ± 0.94 mm at P2, 4.88 ± 0.72 mm at P3, and 5.45 ± 0.77 mm at P4.

**FIGURE 5:**
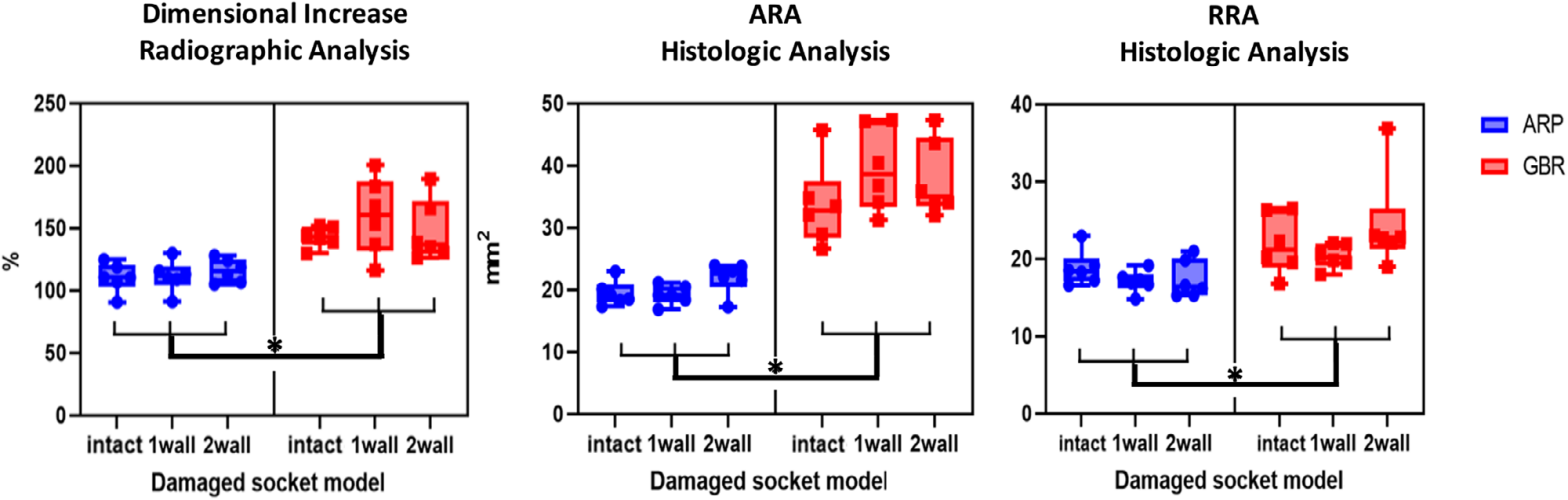
Radiographic and histomorphometric results for dimensional alteration alterations of augmented and regenerated alveolar ridges. Asterisks(^*^) indicate significant differences between ARP group and GBR group, regardless of the extraction‐socket configuration.

In the radiographic analyses, the dimensional increase from baseline was significantly smaller in the ARP group (112.49 ± 10.52%) than in the GBR group (150.03 ± 22.37%, *p *= 0.001), irrespective of the extraction‐socket configuration. The dimensional alterations in the ARP versus GBR groups were 110.33 ± 10.63% versus 142.92 ± 7.50%, 111.39 ± 11.37% versus 159.62 ± 28.02% and 115.77 ± 8.56% versus 147.52 ± 22.60% (*p *= 0.316) in the intact, 1‐wall‐damaged and 2‐walls‐damaged models, respectively (Figure 5).

In the histologic analyses, ARA was significantly smaller in the ARP group (20.16 ± 2.23 mm^2^) than in the GBR group (36.94 ± 6.46 mm^2^, *p *= 0.001). In addition, RRA was significantly smaller in the ARP group (17.73 ± 2.04 mm^2^) than in the GBR group (22.22 ± 4.32 mm^2^, *p *= 0.009), regardless of the extraction‐socket configuration. ARA and RRA in the ARP versus GBR groups were 19.38 ± 1.80 mm^2^ and 18.78 ± 2.05 mm^2^ versus 33.56 ± 6.07 mm^2^ and 21.94 ± 3.55 mm^2^, respectively, in the intact model, 19.17 ± 1.38 mm^2^ and 17.07 ± 1.28 mm^2^ versus 39.49 ± 6.14 mm^2^ and 20.32 ± 1.40 mm^2^ in the 1‐wall‐damaged model, and 21.93 ± 2.25 mm^2^ and 17.35 ± 2.24 mm^2^ versus 37.77 ± 5.66 mm^2^ and 24.40 ± 5.74 mm^2^ in the 2‐walls‐damaged model (*p *= 0.189 and *p *= 0.216) (Figure 5).

BIC was slightly lower in the ARP group (33.79 ± 12.32%) than in the GBR group (40.19 ± 11.88%, *p* = 0.118), but the difference was not statistically significant. BIC values in the ARP versus GBR groups were 29.49 ± 12.82% versus 32.48 ± 10.89%, 30.58 ± 4.55% versus 37.85 ± 7.71% and 41.31 ± 13.59% versus 50.23 ± 8.91% (*p *= 0.801) in the intact, 1‐wall‐damaged and 2‐walls‐damaged models, respectively. Regardless of the treatment group, BIC increased in the following order: intact (30.98 ± 11.99%) < 1‐wall‐damaged (34.21 ± 7.30%) < 2‐walls‐damaged (45.77 ± 12.33%, *p *= 0.010) (Table 1).

**TABLE 1:** Linear measurements related to osseointegration.

Parameter	ARP	GBR	*p*‐value
BIC^b^			
Intact	29.49 ± 12.82	32.48 ± 10.89	0.699
1‐wall‐damaged	30.58 ± 4.55	37.85 ± 7.71	0.100
2‐walls‐damaged	41.31 ± 13.59	50.23 ± 8.91	0.248
Average	33.79 ± 12.32	40.19 ± 11.88	0.118
fBIC^a,b^			
Intact	3.90 ± 1.08	2.72 ± 0.71	0.067
1‐wall‐damaged	4.58 ± 0.57	3.21 ± 0.71	0.007^a^
2‐walls‐damaged	3.23 ± 1.09	1.97 ± 0.64	0.050
Average	3.90 ± 1.09	2.63 ± 0.85	0.012^a^
NBS^a,b,c^			
Intact	3.61 ± 1.04	4.21 ± 0.74	0.320
1‐wall‐damaged	1.73 ± 0.98	3.31 ± 0.89	0.023
2‐walls‐damaged	1.47 ± 1.23	4.76 ± 1.05	0.001^a^
Average	2.27 ± 1.45	4.09 ± 1.08	0.003^a^

*Notes*: Data are mean ± standard deviation values.

^a^Significant difference between the 2 groups regardless of extraction‐socket model type (Average) or for each type of extraction‐socket model (intact, 1‐wall‐damaged and 2‐walls‐damaged models) in repeated‐measures ANOVA (*p *< 0.05) and *t*‐test with Bonferroni post‐hoc analysis (*p *< 0.016).

^b^Significant difference among the 3 socket models in repeated‐measures ANOVA (*p *< 0.05).

^c^Significant difference between the 2 groups and the 3 socket models in repeated‐measures ANOVA (*p *< 0.05).

fBIC was significantly larger and natural bone support was significantly smaller in the ARP group (3.90 ± 1.09 mm and 2.27 ± 1.45 mm, respectively) than in the GBR group (2.63 ± 0.85 mm and 4.09 ± 1.08 mm, *p *= 0.012 and *p *= 0.003), regardless of the extraction‐socket configuration. fBIC and natural bone support in the ARP versus GBR groups were 3.90 ± 1.08 mm and 3.61 ± 1.04 mm versus 2.72 ± 0.71 mm and 4.21 ± 0.74 mm, respectively, in the intact model, 4.58 ± 0.57 mm and 1.73 ± 0.98 mm versus 3.21 ± 0.71 mm and 3.31 ± 0.89 mm in the 1‐wall‐damaged model, and 3.23 ± 1.09 mm and 1.47 ± 1.23 mm versus 1.97 ± 0.64 mm and 4.76 ± 1.05 mm (*p *= 0.939 and *p *= 0.005) in the 2‐walls‐damaged model (Table 1).

## 4 DISCUSSION

This preclinical study comparing alveolar ridge changes and bone regeneration at intact and damaged socket sites after ARP and implant placement versus unassisted socket healing followed by GBR with simultaneous implant placement predominantly revealed:

1. **Superior bone formation and greater bone gains** by GBR compared to ARP, irrespective of the initial socket configuration
2. **Maintenance of the preexisting alveolar ridge dimensions by ARP**, but with some implant dehiscence showing incomplete osseointegration.
3. **Greater augmentation** of the alveolar ridge dimensions by GBR, with entire implant surface surrounded by augmented tissue.

In our prior investigation involving beagle dogs, micro‐CT volume and histomorphometric analyses were conducted at the 16‐week mark following GBR.^29^ In the current study, measurements were taken 8 weeks post‐GBR and 16 weeks post‐ARP. DPBM remodeling with coverage by the non‐crosslinked collagen membrane at 1, 2, 4, 8, and 12 weeks^6^, ^26^, ^30^, ^31^ after ridge augmentation was corroborated by findings from our previous dog studies. We observed a gradual increase in the area of newly formed bone until 4 weeks, followed by a subsequent expansion toward the outermost border until 8 weeks, where it reached a stable state. We utilized non‐crosslinked collagen membrane, which has a degradation time of 12‐16 weeks (as observed in beagle study), to reduce the risk to the newly regenerated tissues and to optimize tissue integration.^32^, ^33^ DPBM is recognized for its biocompatibility, bioabsorbability, and osteoconductivity.^34^ Previous studies of ridge augmentation of damaged extraction sockets using DPBM have confirmed comparable bone formation and post‐operative dimensional shrinkage to that achieved using DBBM.^16^, ^17^, ^35^

The present study revealed superior bone and dimensional gains at sites receiving GBR and simultaneous implant placement compared to sites underwent ARP and implant placement, irrespective of initial socket configuration. The height of residual buccal bone benefits bone gain.^17^ In fact, it is interesting that the differences tended to be more robust when there was a buccal dehiscence. This is explained by clinical tendency to over‐augment during GBR procedures to offset potential bone graft resorption during the healing period.^36^ Conversely, when the socket is intact, bone grafting is primarily performed within the bony envelope, which may explain the less alteration. Furthermore, on another relevant concept, the goal of ARP is to preserve the alveolar ridge rather than to augment it.^15^ Despite that GBR and ARP share a similar therapeutic goal (e.g., adequate ridge dimensions) they are not entirely comparable. While GBR can be performed at various stages and with well‐established principles, ARP is carried out immediately after tooth extraction, and the underlying physiology remains to be elucidated.

ARP has proven to maintain the preexisting alveolar ridge dimensions with strong scientific evidence.^8^, ^9^, ^10^, ^11^, ^28^ The present results for the ARP group are consistent with previous findings of the alveolar ridge maintaining its dimensions comparably in all intact and damaged extraction sockets.^13^, ^14^, ^15^, ^26^ However, it is essential to note that the majority of studies available on ARP have predominantly concentrated on changes in alveolar ridge dimensions until implant placement, overlooking the subsequent changes.^15^

In the present study, sites underwent GBR showed a well‐maintained augmented volume, completely surrounding the dental implants, regardless of the initial socket configuration. In contrast, ARP sites exhibited a dehiscence of buccal bone in both intact or damaged sockets. Although several studies demonstrate stable long‐term outcomes of implants inserted without treating dehiscences,^2^ Often, and despite ARP, additional GBR at implant placement is needed.^18^, ^37^, ^38^, ^39^, ^40^ Therefore, it has been postulated that the additional benefit of ARP depends on the implant placement protocol.^15^, ^18^ Distinctive advantage of GBR is attributed to the secluded space it creates, facilitating bone formation.^41^ GBR, a well‐documented technique apt for horizontal defects,^42^ furthermore proved its efficacy even when fenestrated and peri‐implant buccal bone dehiscences.^43^ Recent studies including RCTs,^44^ have demonstrated volume stability and bone formation within the extensively and horizontally augmented sites even without additional membrane fixation (e.g., pins).^15^, ^29^

Histologic results obtained at GBR sites indicated some bone formation extending beyond the bony envelope. These findings differ from a previous preclinical report suggesting that bone regeneration mainly occurs within the bone envelope, while any residual biomaterial outside the original ridge contour was encapsulated within the connective tissue.^29^ These contrast observations may be attributed to the different observational periods in the 2 experiments: 8 weeks for the present and 16 weeks for the previous one.^29^ Notably, the highly magnified views of the current histologic slides at GBR sites also showed evidence of osteogenesis in the spaces between the biomaterial granules and on their surfaces (Figure 4), suggesting bone regeneration in the coronal region of the augmented area. Together, GBR not only ensures enhanced augmentation and bone regeneration compared to ARP in narrow alveolar ridges but also guarantees full coverage of the implant surface.

In addition, GBR sites at which spontaneous healing was allowed were found to be filled with natural bone at all of the intact and damaged extraction sockets. The histologic findings from conventional ARP studies revealed a wide range of histologic bone regeneration in clinical data,^45^ as well as at damaged socket sites.^17^ Although some data show the feasibility of regenerated bone tissue to support the dental implant,^46^ heterogenic bone regeneration at the ARP sites should be interpreted carefully since the allowed healing period affects the degree of natural bone support.^17^

Bone regeneration is influenced significantly by the defect configuration type at the surgical sites.^47^ The presence of a wall defect increases dimensional shrinkage and the degree of collapse,^6^ moreover prospectively affects the bone quality after the treatment. The natural bone support differed with the number of residual walls in the ARP group. Intact sockets underwent ARP showed more‐favorable natural bone support than did the 1‐wall‐damaged and 2‐wall‐damaged sockets. This pattern resembles the dimensional healing being affected by the range of periodontally compromised extraction sockets, which confirms previous findings.^47^

ARP technique is a simplified approach aimed at reducing patient morbidity compared to GBR, renowned for its shorter treatment time and minimal invasiveness.^48^ Nonetheless, it necessitates additional bone grafting in approximately 10% of the cases.^49^ Randomized clinical trials have demonstrated comparable patient‐reported outcome measurements for implant placement with or without ARP.^18^ However, in the absence of preoperative notification, unexpected increases in costs and treatment duration may arise. On the other hand, a significant drawback of the GBR procedure lies in its invasiveness, necessitating vertical incisions and releasing periosteoum.^50^ The current study suggests a potential necessity for additional bone grafting in cases of narrow alveolar ridges, with GBR tentatively preferred over ARP in such instances.

The study has limitations. First, ARP sites underwent flap elevation prior to implant placement, potentially leading to a greater loss of augmented biomaterials compared to GBR sites. Second, in the ARP group, implant dehiscence was left untreated without additional bone grafting during the second surgery to maintain consistent conditions across experimental sites. It's important to note that, in clinical settings, the coverage of exposed implant surfaces is typically performed additionally. Third, over‐augmentation was not performed for the ARP sites. The study adhered to the most documented concept of ARP which is to preserve the original alveolar ridge dimensions, rather than extending beyond the bony envelope. Different approaches may be applied in the clinical scenario including the application of stabilization devices to allow grafting beyond the bony envelope.

## 5 CONCLUSION

Early implant placement with ARP mitigates dimensional changes in the narrow alveolar ridge. However, simultaneous GBR creates conditions for enhanced bone regeneration around the implant and greater ridge augmentation compared to ARP, irrespective of the extraction‐socket configuration.

## AUTHOR CONTRIBUTIONS

All authors have made substantial contributions to this study. H.J.S analyzed the data and wrote the paper; J.Y.P, F.J.S. J.K.C critically reviewed and revised the manuscript; H.K.T and J.S.L performed experiment and collected the data. J.S.L conceived and designed the whole experiment and approved the article submission.

## CONFLICT OF INTEREST STATEMENT

The authors declare no conflicts of interest.

## Data Availability

The data that support the findings of this study are available from the corresponding author upon reasonable request.
